# Smoking and environmental characteristics of smokers with a mental illness, and associations with quitting behaviour and motivation; a cross sectional study

**DOI:** 10.1186/s12889-016-2969-1

**Published:** 2016-04-14

**Authors:** Alexandra P. Metse, John Wiggers, Paula Wye, Lyndell Moore, Richard Clancy, Luke Wolfenden, Megan Freund, Tara Van Zeist, Emily Stockings, Jenny A. Bowman

**Affiliations:** University of Newcastle, University Drive, Callaghan, NSW 2308 Australia; Hunter Medical Research Institute, Lot 1 Kookaburra Circuit, New Lambton Heights, NSW 2305 Australia; Hunter New England Population Health, Longworth Ave, Wallsend, NSW 2287 Australia; Centre for Translational Neuroscience and Mental Health, Corner Edith and Platt Streets, Waratah, NSW 2298 Australia; National Drug and Alcohol Research Centre, University of New South Wales, 22-32 King Street, Randwick, NSW 2031 Australia

**Keywords:** Smoking, Mental illness, Characteristics, Social, Environment, Quitting, Quitting motivation

## Abstract

**Background:**

Persons with a mental illness are less likely to be successful in attempts to quit smoking. A number of smoking and environmental characteristics have been shown to be related to quitting behaviour and motivation of smokers generally, however have been less studied among smokers with a mental illness. This study aimed to report the prevalence of smoking characteristics and a variety of physical and social environmental characteristics of smokers with a mental illness, and explore their association with quitting behaviour and motivation.

**Methods:**

A cross-sectional descriptive study was undertaken of 754 smokers admitted to four psychiatric inpatient facilities in Australia. Multivariable logistic regression analyses were undertaken to explore the association between smoking and environmental characteristics and recent quitting behaviour and motivation.

**Results:**

Participants were primarily daily smokers (93 %), consumed >10 cigarettes per day (74 %), and highly nicotine dependent (51 %). A third (32 %) lived in a house in which smoking was permitted, and 44 % lived with other smokers. The majority of participants believed that significant others (68–82 %) and health care providers (80–91 %) would be supportive of their quitting smoking. Reflecting previous research, the smoking characteristics examined were variously associated with quitting behaviour and motivation. Additionally, participants not living with other smokers were more likely to have quit for a longer duration (*OR* 2.02), and those perceiving their psychiatrist to be supportive of a quit attempt were more likely to have had more quit attempts in the past six months (*OR* 2.83).

**Conclusions:**

Modifiable characteristics of the physical and social environment, and of smoking, should be considered in smoking cessation interventions for persons with a mental illness.

## Background

A decreased likelihood of successfully quitting [1, 2] contributes to the persistently higher prevalence of smoking among persons with a mental illness [3–6]. This in turn is reflected in greater morbidity and mortality from tobacco-related diseases and a reduced life expectancy [7, 8].

Successful smoking cessation is often preceded by multiple quit attempts [9, 10], a motivation to quit [9, 11], and a greater capacity to identify as a potential non-smoker [12–14]. The likelihood of being motivated and attempting to quit has been suggested to be a function of an individual’s smoking characteristics such as level of nicotine dependence [15, 16], number of cigarettes smoked per day [17, 18], age of smoking initiation [10, 19], and number of years smoking [20]. While research among smokers with a mental illness specifically has consistently demonstrated high levels of nicotine dependence and heavy smoking (in terms of cigarettes per day) [3, 5, 6], far less research has explored the associations of such characteristics with quitting behaviour and motivation. That which has been undertaken however suggests the same patterns of association as found among smokers generally [11, 21, 22]. For instance, lower levels of nicotine dependence have been associated with quit attempts of longer duration (>7 days) among smokers from the United States (U.S.) attending a residential substance treatment program [22], and following discharge from a psychiatric inpatient facility [11].

Socio-ecological theories also highlight the potential influence of a range of broader physical and social environmental characteristics of smokers on quitting behaviour [23]. Among smokers generally, characteristics associated with both attempts to quit and successful cessation include: residing in a smoke-free home [10, 24, 25]; absence of persons who smoke in the immediate environment [10, 25]; a partner being a non-smoker [26, 27]; and perception of support to quit from significant others [28, 29] and health care providers [16, 30]. Consequently, intervention strategies to aid smoking cessation have included: the introduction of smoke-free workplace policies [31]; enhancement of partner support for spouse’s smoking cessation [32]; smoking cessation care being delivered by health care providers [33]; and increasing the availability of smoking cessation peer support programs [34].

Very little research has explored the prevalence of such physical and social environmental characteristics among persons with a mental illness. In the U.S., a study reported that only 24 % of smokers diagnosed with psychotic or mood disorders occupied a residence where smoking was not permitted inside [35], nearly half that reported in a national representative sample of smokers without a mental illness (46 %) [36]. Among clients of U.S. community mental health services, the proportion of smokers with acquaintances that smoked was more than twice that of non-smokers (78 % vs 35 %) and smokers were reported to have higher rates of household second-hand smoke exposure (47 % vs 28 %) [37]. In one Australian study, the proportion of smokers with a mental illness living in a smoke-free home was found to be 42 %, almost 20 % less than those without a mental illness [38].

In addition, few studies have explored the association between the physical and social environmental characteristics of smokers with a mental illness and their quitting behaviour and motivation. In one such study, among U.S. smokers with co-occurring severe mental illness and substance use disorders, Ferron et al. [39] found that more social contact with non-substance using (including tobacco) friends was positively associated with a higher number of quit attempts. Survey data from the United Kingdom (UK) [40] and U.S. [41] similarly suggest that aspects of the physical and social environment have an impact on smoking and quitting behaviour of smokers with a mental illness. Among recent quitters with a current mental illness, 46–58 % identified social support to quit from friends, family and doctors as key enabling factors in their successful quitting [41]; and among forensic psychiatric inpatients, exposure to others smoking and lack of encouragement from psychiatric staff to quit were identified as barriers to successful smoking cessation [40]. Further, qualitative research undertaken in several countries, including g the U.S. [42, 43], UK [44], Scotland [45], Australia [46], and Canada [47], has suggested that a lack of support to quit from family and friends [44] and health care professionals [43–46], and socialising with other smokers [42, 45, 47] contribute to continued smoking by this group.

Given the limited evidence available, it is suggested that a greater understanding of the characteristics of smokers with a mental illness that are associated with their quitting behaviour and motivation [48] is required to facilitate the development of effective smoking cessation interventions. We conducted a study that aimed to 1) report the prevalence of smoking characteristics and a variety of physical and social environmental characteristics of smokers with a mental illness and 2) explore the association between such characteristics and recent quitting behaviour and motivation.

## Methods

### Design and setting

A cross-sectional descriptive study was undertaken in the context of a smoking cessation intervention trial [49] conducted in four adult psychiatric inpatient facilities in New South Wales (NSW), Australia.

### Sample and recruitment procedure

Research staff approached all patients admitted to the four psychiatric inpatient facilities over a 19 month period (October 2012 and April 2014) to assess for study eligibility [49]. Research staff were independent of the hospitals, received standardised training in mental illness and its impacts, and had completed or were  in the process of completing an undergraduate degree in a health related area. Patients eligible for the trial were: current smokers (smoked tobacco in the month prior to admission); at least 18 years of age; willing to provide contact details; and able to give informed consent to participate in the trial. No other exclusion criteria were applied.

### Ethics, consent and permissions

Ethics approval was obtained from the Hunter New England Human Research Ethics Committee (reference no: 11/12/14/4.02) and the University of Newcastle Human Research Ethics Committee (reference no: H-2012-0061). Written consent was obtained from all participants.

### Data collection procedures

Consenting participants completed a face-to-face structured interview, administered by research staff during their hospital stay. Interviews were administered in a quiet area of the inpatient unit and took approximately 40 min to complete. Participants could opt to have short breaks during the data collection process as required. Interviews were carried out prior to participant allocation to the intervention or usual care control condition of the overarching smoking trial [49]. Characteristics of smoking and of the participants’ physical and social environment relevant to smoking, and recent quitting behaviour and motivation were collected by the interview. Participant clinical and demographic information was obtained via the facility electronic medical record system and the participant interview.

### Measures

#### Clinical and demographic information

The following participant data were collected from the patient medical record system: age, gender, relationship status (single, married/de facto, separated/divorced, widowed, did not state/inadequately described), Aboriginal and/or Torres Strait Islander status (Aboriginal and/or Torres Strait Islander, neither, did not state), primary mental health diagnosis at discharge (schizophrenia and related psychoses, anxiety and stress related disorders, mood disorders, substance- related disorders, personality and other disorders), legal status at admission (voluntary, involuntary), and length of stay (total days between admission and discharge).

The following clinical and demographic information was obtained from the participant interview: level of alcohol use (AUDIT-C) [50], education (primary school, third year of high school, school certificate (fourth year high school), Higher School Certificate [HSC] (sixth year high school), TAFE certificate or diploma (tertiary qualification not obtained from a university), bachelor degree, post graduate degree), employment details (full time, part time, household duties, student, unemployed/other), receipt of a government payment (yes, no), and living circumstances (on own, with others).

#### Smoking characteristics

The smoking characteristics of participants prior to admission were: smoking status (daily smoker, weekly smoker, irregular smoker [smoked cigarettes less than weekly), cigarettes per day, level of nicotine dependence (Fagerstrom Test for Nicotine Dependence [FTND]) [51], age initiated smoking, and number of years smoked.

#### Physical and social environmental characteristics

Participants were asked if they: lived in a smoke-free house prior to admission (a place of residence where smoking is not permitted inside; yes, no), lived with smokers prior to admission (lived with at least one other smoker; yes, no), and had a partner who smoked (partner smokes, partner does not smoke/no partner).

Participants were also asked to rate on a seven point Likert- type scale (‘very supportive’ to ‘actively discourage’, ‘unsure’ and ‘not applicable’), how supportive they perceived the following persons would be if they were to attempt to quit smoking: partner, family, friends, general practitioner (primary care physician; GP), psychiatrist, and ‘other’ mental health professional (psychologists, social workers, nurses, counsellors and case managers). Participants were asked if they had someone in their life they felt was their key support person (a person of whom they could rely and/or routinely provided assistance and general support; yes, no).

#### Recent quitting behaviour and motivation

Measures of recent quitting behaviour were: a quit attempt in past six months (yes, no); and, for those who had attempted to quit in the past six months, the number of times (once, two to three times, more than three times) and duration of longest quit attempt in that period (days). Quit attempts were defined as not smoking on purpose for a period of at least 24 h, with the intention of quitting smoking [52].

Measures of smoking related motivation were: readiness to quit and smoking identity. To assess current readiness to change, the Readiness to Quit Smoking Questionnaire [53] was used, eliciting responses to five items in a Likert scale format. Smoking identity was measured using a single question asking respondents to indicate how easy it was for them to see themselves as a non-smoker [14]. Participants responded on a five point Likert- type scale ranging from very easy to very difficult, with an ‘unsure’ option.

### Analysis

Data were analysed using SPSS Statistics version 22 [54]. The following numerical variables were transformed to categorical variables for the purpose of association analyses: cigarettes per day (1–10, 11–20, 21–30, >31) [55], age initiated smoking (<14 years, ≥ 14 years) [56], number of years smoked (≤10, 11–20, >20 years) [20], number of quit attempts in the past six months (1, ≥2) [9, 57], and duration of longest quit attempt in the last six months (<1 month, ≥ 1 month) [10, 57].

The following variables were categorised to two levels: smoking status (daily smoker, weekly/irregular smoker), nicotine dependence (low-moderate [FTND score ≤5], high [FTND score ≥ 6]) [58], readiness to quit (pre-contemplative, contemplative or a more progressed stage), and ease of seeing self as a non-smoker (easy, difficult/unsure). All measures pertaining to the degree of perceived social support to quit smoking from significant persons/clinicians were also categorised to two levels (supportive, unsupportive/unsure/not applicable).

Participant clinical and demographic information, smoking and physical and social environmental characteristics, and recent quitting behaviour and motivation were summarised using descriptive statistics.

Chi-square analysis was used to explore univariate associations between each separate smoking and physical and social environmental characteristic, and recent quitting behaviour and motivation. Variables with a *p*-value of ≤ 0.25 were subsequently entered into multivariable logistic regression models, using both backward elimination and stepwise variable selection to ensure model stability. Significance level was set at 0.05 for the inclusion of variables in the final models. Separate models were developed for five dependent variables: quit attempt in the past six months (yes, no), number of quit attempts in past six months (one, two or more), duration of quit attempt in the past six months (<1 month, ≥ 1 month), readiness to quit (pre-contemplative, contemplative or a more progressed stage), and ease of seeing self as a non-smoker (easy, difficult/unsure).

## Results

### Sample

**Fig. 1:**
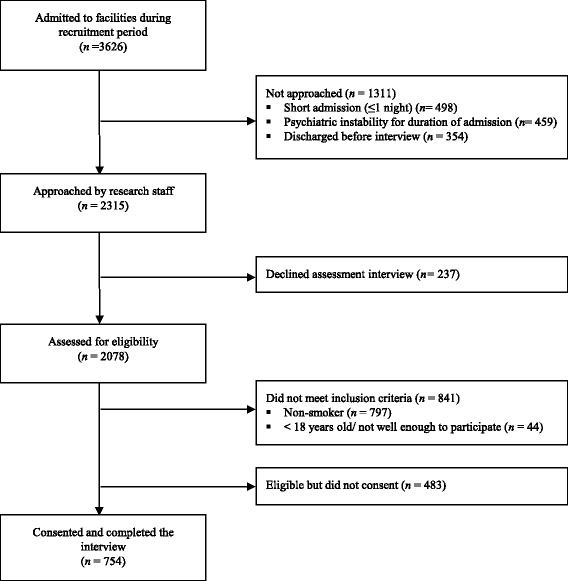
Flow diagram illustrating the number of patients approached, assessed for eligibility and recruited into the study

Of the 3626 patients admitted to the four inpatient facilities in the study period, 64 % (*n* =2315) were approached by research staff. For those not approached, the primary reasons for non-contact were a short length of stay (≤ one night; 38 %) and psychiatric instability for the duration of time spent as an inpatient (35 %). Of the 2315 patients approached, 2078 (90 %) agreed to be assessed for study eligibility, of which 841 (40 %) were ineligible, predominantly due to being non-smokers (*n* = 797, 95 %). Sixty one per cent (*n* = 754) of eligible smokers consented and completed the survey (Fig. 1).

### Patient clinical and demographic information

Table 1 describes the clinical and demographic characteristics of patients approached and not approached, and participants and non-consenters. Approximately one third (30 %) of participants lived on their own, 66 % with a partner or with others, and 4 % reported being homeless (not in table).

**Table 1:** Clinical and demographic characteristics of not approached and approached patients, and participants and non-consenters

	Not approached (*N* = 1311)	Approached (*N* = 2315)	Non-consenters (*N* = 483)	Participants (*N* = 754)
Gender (%)				
Male	60.0	55.4	63.4	61.3
Female	40.0	44.6	36.6	38.7
Age (years)				
Mean (SD)	39.8 (17.1)	41.8 (14.2)	38.9 (11.7)	38.7(12.0)
Median (range: min-max)	37 (10–94)	41 (18–93)	38 (18–82)	38 (18–76)
Relationship status (%)				
Single	59.0	58.6	70.8	63.7
Married/de facto	25.7	24.1	17.4	20.7
Separated/divorced/widowed	14.2	16.5	10.8	15.0
Not stated/inadequately described	1.0	0.7	1.0	0.7
Aboriginal and/or Torres Strait Islander Status (%)				
Aboriginal and/or Torres Strait Islander	12.8	11.6	17.7	13.5
Neither Aboriginal or Torres Strait Islander/unknown	87.2	88.4	82.3	86.5
Employment status (%)				
Full time	–	–	–	15.1
Part time	–	–	–	11.4
Student	–	–	–	2.8
Unemployed/household duties/other	–	–	–	70.7
Highest education level achieved (%)				
Up to third year of high school	–	–	–	28.4
School Certificate	–	–	–	32.6
Higher School Certificate (HSC)	–	–	–	13.4
Tertiary	–	–	–	25.6
Receipt of a government payment (%)				
Yes	–	–	–	77.1
No	–	–	–	22.9
Diagnosis type (%)				
Schizophrenia and related psychosis	14.1	27.6	37.1	19.5
Anxiety and stress related disorders	20.3	8.5	6.4	13.0
Mood disorders	23.1	30.8	22.4	26.7
Substance related disorders	21.2	15.6	18.0	23.1
Personality and other disorders	21.3	17.4	16.1	17.8
Alcohol use (AUDIT- C) (%)*				
Harmful/hazardous	–	–	–	64.5
Non-harmful/hazardous	–	–	–	35.5
Length of stay (days)				
Mean (SD)	12.4 (62.1)	16.8 (28.7)	17.6 (24.4)	14.3 (17.9)
Median (Range: min-max)	2 (0–1715)	10 (0–945)	10 (0–236)	9 (0–147)
Legal status on admission (%)				
Voluntary	55.6	53.2	49.3	53.2
Involuntary	44.4	46.8	50.7	46.8

### Prevalence of smoking characteristics

Participants smoking characteristics are described in Table 2. Almost all (93 %) were daily smokers; 51 % were assessed as highly nicotine dependent; nearly three quarters (74 %) smoked >10 cigarettes per day; 68 % had commenced smoking before 16 years of age; and more than half (55 %) had smoked for more than 20 years.

**Table 2:** Smoking characteristics

	Total (*N* = 754)
Smoking status (%)^a, b, c, d, e^	
Daily	93.0
Weekly	3.6
Irregular	3.4
Cigarettes Per Day (%)^a, b, c, e^	
1–10	26.1
11–20	36.2
21–30	23.2
> 30	14.5
Level of nicotine dependence (%)^a,c, e^	
Low-moderate	48.8
High	51.2
Age initiated smoking (%)^d, e^	
< 12	21.8
12 - < 14	21.0
14 - < 16	25.2
16 - < 18	16.2
≥ 18	15.9
Number of years smoked (%)^a, b, c, d, e^	
≤ 10	18.3
11–20	26.9
> 20	54.8

Entered into regression analyses: ^a^Quit attempt in the past 6 months; ^b^Number of quit attempts in the past 6 months; ^c^Length of longest quit attempt in past 6 months; ^d^Readiness to quit; ^e^Ease of seeing oneself as a non-smoker

### Prevalence of physical and social environmental characteristics

Forty four per cent of participants lived with at least one other smoker, and 32 % lived in a house where smoking was permitted inside (Table 3). Fifty one per cent of those with a partner reported that their partner smoked.

**Table 3:** Physical and social environmental characteristics

	% (*n*)^a^
Lived with smokers (%)^d,e^	
Yes	43.6 (329)
No	56.4 (425)
Lived in a smoke-free house (%)^d^	
Yes	68.0 (513)
No	32.0 (241)
Partner smoking status (%)^e^	
Partner smokes	50.6 (79)
Partner does not smoke	49.4 (77)
Identify a key support person (%)	
Yes	92.4 (697)
No	7.6 (57)
Perceived social support to quit smoking from:	
Partner (%)^d, e^	
Supportive	71.2 (111)
Unsupportive	28.2 (44)
Unsure	0.6 (1)
Family (%)^f^	
Supportive	82.3 (552)
Unsupportive	8.9 (60)
Unsure	8.8 (59)
Friends (%)^e^	
Supportive	68.0 (433)
Unsupportive	13.1 (84)
Unsure	18.8 (120)
Psychiatrist (%)^c^	
Supportive	80.9 (408)
Unsupportive	7.0 (35)
Unsure	12.1 (61)
General Practitioner (%)^c^	
Supportive	91.4 (601)
Unsupportive	2.9 (19)
Unsure	5.8 (38)
Other mental health professional (%)^c^	
Supportive	80.2 (469)
Unsupportive	8.5 (50)
Unsure	11.3 (66)

^a^Total numbers vary due to applicability

Entered into regression analyses: ^b^Quit attempt in the past 6 months; ^c^Number of quit attempts in the past 6 months; ^d^Length of longest quit attempt in past 6 months; ^e^Readiness to quit; ^f^Ease of seeing oneself as a non-smoker

Almost all participants (92 %) could identify a key support person in their life on whom they could rely and/or routinely provided assistance and general support. Seventy one per cent of participants with a partner believed the partner would be supportive of them making a quit attempt. Eighty two per cent and 68 % of participants believed their family and friends would be supportive of them quitting, respectively. Eighty one, 91 and 80 % of participants believed that their psychiatrist, GP, or another mental health professional, respectively, would be supportive of them attempting to quit.

### Quitting behaviour and motivations

Thirty one per cent of participants had attempted to quit in the past six months; with 57 % of those who had done so having made a single quit attempt, and 30 and 13 % having attempted to quit two to three times or more than three times, respectively. Of those who had made a quit attempt in the past six months, 21 % were abstinent for at least one month.

In terms of motivation and readiness to quit, 45 % of participants were assessed as being either contemplative or at a more progressed stage. Forty three percent reported it would be easy to see themselves as a non-smoker, whereas 43 and 14 % reported it would be difficult or that they were unsure.

### Smoking, physical and social and environmental characteristics associated with quitting behaviour and motivation

Variables with a *p*-value of ≤0.25 in the chi-square analyses and hence entered in the multivariable logistic regression models are noted in Tables 2 and 3. The findings of the five regression models were as follows:

- *Quit attempt in the past six months:* Weekly/irregular smokers were twice as likely as daily smokers to have attempted to quit in the past six months (*OR* = 2.07, 95 % confidence interval (CI): 1.17 to 3.64, *p* < 0.05) (Table 4). Those who had been smoking for less than 10 years were 1.65 (95 % CI: 1.11 to 2.47, *p* < 0.05) times more likely than those who had smoked for more than 20 years to have attempted to quit in the past six months.
- *Number of quit attempts in the past six months:* Smokers who perceived their psychiatrist to be supportive of them quitting smoking were 2.83 (95 % CI: 1.64 to 4.88, *p* < 0.001) times more likely than those who perceived their psychiatrist to be unsupportive or who did not have a psychiatrist to have attempted to quit two or more times in the past six months.
- *Duration of quit attempt in the past six months:* Smokers of 1–10 cigarettes per day were 16.23 (95 % CI: 2.05 to 128.24, *p* < 0.05) times more likely to have made a quit attempt of longer duration, compared to heavier smokers (31 cigarettes or more). Participants not residing with other smokers were 2.02 (95 % CI: 1.002 to 4.06, *p* < 0.05) times more likely to have quit for one month or longer in the past six months.
- *Readiness to quit:* Participants who started smoking at the age of 14 or after were 1.40 (95 % CI: 1.04 to 1.87, *p* < 0.05) times more likely be in the contemplative or a more progressed stage of change, relative to those who initiated smoking prior to the age of 14.
- *Identity as a smoker:* Weekly/irregular smokers were 2.79 (95 % CI: 1.47 to 5.29, *p* < 0.05) times more likely than daily smokers to easily see themselves as a non-smoker; low-moderate nicotine dependent smokers were 2.34 (95 % CI: 1.72 to 3.18, *p* < 0.001) times more likely to easily see themselves as a non-smoker than highly dependent smokers; and participants who had been smoking for less than 10 years were 2.34 (95 % CI: 1.55 to 3.52, *p* < 0.001) times more likely to easily see themselves this way than participants who had smoked for more than 20 years.

**Table 4:** Multivariable logistic regression results for smoking and physical and social environmental characteristics associated with quitting behaviour and motivation

Predictor	% (*n*)	OR	95 % CI	
			Lower	Upper
Model 1: Quit attempt in the past 6 months^a^				
Smoking status				
Weekly/Irregular	7.0 (53)	2.07*	1.17	3.64
Daily	93.0 (701)	Ref		
Years smoked				
≤ 10	18.3 (138)	1.65*	1.11	2.47
11–20	26.9 (203)	0.99	0.68	1.43
> 20	54.8 (413)	Ref		
Model 2: Number of quit attempts in the past 6 months^b^				
Perceived support from psychiatrist				
Supportive	54.7 (128)	2.83**	1.64	4.88
Unsupportive/not applicable	45.3 (106)	Ref		
Model 3: Duration of quit attempt in the past 6 months^c^				
Lived with smokers				
No	56.4 (132)	2.02*	1.002	4.06
Yes	43.6 (102)	Ref		
Cigarettes per day				
1–10	32.9 (77)	16.23*	2.05	128.24
11–20	34.2 (80)	4.23	0.52	34.61
21–30	21.8 (51)	5.88	0.70	49.50
31+	11.1 (26)	Ref		
Model 4: Readiness to quit^d^				
Age initiated smoking				
≥ 14 years old	57.3 (432)	1.40*	1.04	1.87
< 14 years old	42.7 (322)	Ref		
Model 5: Identity as a smoker^e^				
Smoking status				
Weekly/Irregular	7.0 (53)	2.79*	1.47	5.29
Daily	93.0 (701)	Ref		
Nicotine dependence				
Low- moderate	48.8 (368)	2.34**	1.72	3.18
High	51.2 (386)	Ref		
Number of years smoked				
≤ 10	18.3 (138)	2.34**	1.55	3.52
11–20	26.9 (203)	1.24	0.87	1.77
> 20	54.8 (413)	Ref		

^a^(reference: no); ^b^(reference: one quit attempt; only participants who had attempted to quit were included); ^c^(reference: < 1 month; only participants who has attempted to quit were included); ^d^(reference: precontemplative); ^e^(reference: difficult to see self as non-smoker); *p < 0.05, ** *p* <0.001; Ref: reference category

## Discussion

This is the first study reporting both the prevalence of a range of smoking and physical and social environmental characteristics of smokers with a mental illness, and the association of such characteristics with quitting behaviour and motivation. In line with previous research, assessment of smoking characteristics indicated a high prevalence of daily smoking, high nicotine dependence and heavy smoking in terms of cigarettes per day; and further, that smoking appeared to be a long-established behaviour. With respect to physical environmental characteristics, smoking was ‘present’ in the home lives of many participants: nearly one half lived with others who were also smokers and for one third, the home was not smoke-free. Whilst with respect to social environmental characteristics, perceived support for quitting from significant others and health professionals was variable, with a lack of support most evident for friends; one third (32 %) not indicating that friends would be supportive.

The results suggest that despite many participants expressing interest in quitting and making recent quit attempts, reflecting the findings of previous research [2, 12], a number of smoking and physical and social environmental characteristics may serve to sustain their tobacco use. In line with the broader smoking literature [15, 16] as well as previous research among smokers with a mental illness [11, 22], ‘lighter’ smokers (less nicotine dependent, smoking fewer cigarettes or less than daily) were more likely to have attempted or to be at least contemplating quitting. Such findings suggest a potential benefit of interventions aimed at reducing cigarette consumption among persons with a mental illness in not only decreasing the degree of immediate harm caused by heavy smoking [59] but also increasing the likelihood of subsequent quit attempts and cessation [4, 60]. The findings that those who had been smoking for a fewer number of years were more likely to envisage life without smoking and to have recently attempted to quit, and that smokers who initiated smoking at a younger age were likely to be less ‘ready’ to quit, similarly reflects findings from general population smokers [10, 19] and perhaps highlights the importance of providing cessation intervention to young smokers and to all smokers as soon as possible after initiation.

In terms of the physical environment, previous research has reported the presence of smoking in the home environment to be less prevalent among the general Australian population, than for the participants in this study: 34 % [61] (as compared to 44 %) living with another smoker, and only 7 % [62] (as compared to 32 %) residing in a home that was not smoke free. Further, differences between Australian smokers without a mental illness are also evident in comparison to smokers in the present study: 21 % residing in a home that was not smoke free (as compared to 32 %), and 42 % of those with a current partner reporting their partner to be a smoker (as compared to 51 %) (International Tobacco Control Policy Evaluation Study: Survey Data, unpublished 2013 and 2014). In line with research undertaken with smokers generally [24, 26, 56], the potential influence of other smokers in the immediate environment was indicated by the finding in this study that participants who did not live with other smokers were more likely to have recently made a quit attempt of at least one month duration.

With respect to the social environment, almost all participants (92 %) identified a key support person whom they relied on and/or who routinely provided assistance and support for their well-being and functioning; suggesting the potential to involve support persons, and possibly family carers [63] in smoking cessation interventions. While evidence suggests that involvement of family carers in treatment delivery can be effective in improving mental health outcomes for persons with a mental illness [64], the potential of such carers to support someone with a mental illness to quit smoking appears not to have been explored by research. One U.S. survey of recent quitters with a mental illness does suggest however, that family and friendship networks generally could have a role in encouraging and supporting quitting behaviour [41]. In the present study, while a majority of participants perceived that family, a partner and friends would be supportive of their making a quit attempt, nevertheless quite significant proportions also reported either that such people would be unsupportive of a quit attempt or that they were uncertain of their support (family 18 %; partner 29 % and friends 32 %), indicating a need for further research to explore their potential to play a role in smoking cessation interventions for this group.

The large majority of participants perceived their GP (91 %), psychiatrist (81 %) and other mental health professional/s (80 %) to be supportive of a quit attempt. Physician advice has been identified to have a particularly positive impact on smoking and quitting behaviour [65] and this was reinforced by the study finding that those participants who perceived psychiatrists as supportive of quitting were more likely to have recently made a greater number of quit attempts. Although the differences in the perceived support across the health professional groups were minimal, ‘other’ mental health professionals were least likely to be seen as supportive; a finding somewhat reflective of previous research suggesting allied health professionals, including psychologists are less likely to routinely assess for or offer intervention for smoking [66], despite the likely efficacy of their doing so [33]. It is possible that higher rates of nicotine dependence reported among some mental health professions, compared to their general health counterparts contributes to a more benign perception of smoking [67] and hence a lower likelihood of intervening. Given the integral role of mental health professionals in caring for persons with a mental illness, and the professional status in particular of psychiatrists, the importance of their accepting provision of smoking cessation care as part of their professional role has been noted previously [68].

The strengths of this study include its conduct with a large and diverse sample of smokers with a mental illness and a relatively high consent rate. However, it is noted that smokers who stayed in the hospital for one night or less and those with psychotic type disorders may be underrepresented, while patients with anxiety/stress and substance-related disorders may be over-represented as compared to aggregate descriptive statistics for the facility’s patient population during the recruiting period. The sample consisted of smokers who had consented to take part in an overarching smoking trial and it is unknown whether the prevalence and role of physical and social environmental factors may have differed somewhat for this sample as compared to the broader group of smokers with a mental illness. It is noted that findings need to be interpreted in the context of the cross sectional study design. Future research employing a longitudinal design would add strength to the conclusions that could be drawn. The utilisation of solely self-report data may also pose a limitation, in that accounts of recent quitting behaviour may have been under or over-estimated.

## Conclusions

This paper provides evidence for the importance of considering characteristics of smoking and also of the physical and social environment in cessation interventions for persons with a mental illness. With respect to the latter, it expands previous knowledge in this field in identifying the importance of encouraging physical environments that promote smoking cessation and the potential benefit of engaging significant others and health care providers, particularly psychiatrists.

## Availability of supporting data

The datasets generated and analysed during the current study are not publicly available to preserve the privacy of participants, however are available from the corresponding author on reasonable request.
